# Supplementary material for: OmicVerse: a framework for bridging and deepening insights across bulk and single-cell sequencing
Source: Nat Commun. 2024 Jul 16;15:5983. doi: 10.1038/s41467-024-50194-3 (PMC11252408; doi:10.1038/s41467-024-50194-3)
Supplement: Supplementary file 3 — Reporting Summary [file 41467_2024_50194_MOESM3_ESM.pdf]

nature portfolio

Corresponding author(s): Zehua Zeng, Cencan Xing, Yuanyan Xiong, Hongwu Du

Last updated by author(s): Jun 17, 2024

Reporting Summary

Nature Portfolio wishes to improve the reproducibility of the work that we publish. This form provides structure for consistency and transparency in reporting. For further information on Nature Portfolio policies, see our Editorial Policies and the Editorial Policy Checklist.

Statistics

For all statistical analyses, confirm that the following items are present in the figure legend, table legend, main text, or Methods section.

n/a	Confirmed	
☐	☒	The exact sample size (*n*) for each experimental group/condition, given as a discrete number and unit of measurement
☐	☒	A statement on whether measurements were taken from distinct samples or whether the same sample was measured repeatedly
☐	☒	The statistical test(s) used AND whether they are one- or two-sided<br>*Only common tests should be described solely by name; describe more complex techniques in the Methods section.*
☒	☐	A description of all covariates tested
☐	☒	A description of any assumptions or corrections, such as tests of normality and adjustment for multiple comparisons
☐	☒	A full description of the statistical parameters including central tendency (e.g. means) or other basic estimates (e.g. regression coefficient) AND variation (e.g. standard deviation) or associated estimates of uncertainty (e.g. confidence intervals)
☐	☒	For null hypothesis testing, the test statistic (e.g. *F*, *t*, *r*) with confidence intervals, effect sizes, degrees of freedom and *P* value noted<br>*Give P values as exact values whenever suitable.*
☒	☐	For Bayesian analysis, information on the choice of priors and Markov chain Monte Carlo settings
☒	☐	For hierarchical and complex designs, identification of the appropriate level for tests and full reporting of outcomes
☐	☒	Estimates of effect sizes (e.g. Cohen's *d*, Pearson's *r*), indicating how they were calculated

Our web collection on statistics for biologists contains articles on many of the points above.

Software and code

Policy information about availability of computer code

Data collection	Data collection was performed using custom code available at https://github.com/Starlitnightly/omicverse-reproducibility/tree/main/docs
Data analysis	OmicVerse analysis was performed using custom Python (v3.9.1) package omicverse (v1.5.3) (available at https://github.com/Starlitnightly/omicverse). Bulk RNA-seq methods were performed using software package pyDEseq2 (v0.3.5), scipy (v1.9.1), scanpy (v1.9.3), gseapy (v0.10.8), dynamicTreeCut (v1.0.0). Single cell RNA-seq methods were performed using software package SEACells (v0.3.3), VIA (v0.1.86), CellphoneDB (v4.0.0), SCSA (v1.0.0). Multi-omics analysis were performed using software package mofax (v0.3.6), mofapy2 (v0.7.0), scglue (v0.3.2). Full environment configuration files canalso be found at https://github.com/Starlitnightly/omicverse

For manuscripts utilizing custom algorithms or software that are central to the research but not yet described in published literature, software must be made available to editors and reviewers. We strongly encourage code deposition in a community repository (e.g. GitHub). See the Nature Portfolio guidelines for submitting code & software for further information.

Data

Policy information about availability of data

All manuscripts must include a data availability statement. This statement should provide the following information, where applicable:

- Accession codes, unique identifiers, or web links for publicly available datasets
- A description of any restrictions on data availability
- For clinical datasets or third party data, please ensure that the statement adheres to our policy

All the datasets used in this study, including five scRNA-seq, four bulk RNA-seq, one snRNA-seq and snATAC-seq are summarized in Suppmentary Note 4,5,6 and are

urated in the omicverse-reproducibility (https://github.com/Starlitnightly/omicverse-reproducibility). They can be easily downloaded and imported directly to reproduce the analyses presented in this manuscript. We have also deposited all the datasets to figshare at https://doi.org/10.6084/m9.figshare.23165636 and https://doi.org/10.6084/m9.figshare.26023324.

In addition, we also provided the source of these published datasets. The Dentate Gyrus data used in this study have been deposited in the Gene Expression Omnibus (GEO) database under accession code GSE95753 and GSE74985, Data related to pancreatic endocrinogenesis are accessible via accession codes GSE132188 and GSE189434, the maturation of murine liver data can be found under accession code GSE171993 and GSE58827, Human bone marrow data are available in the Human Cell Atlas (HCA) database at https://data.humancellatlas.org/explore/projects/091cf39b-01bc-42e5-9437-f419a66c8a45 and in the GEO database under accession code GSE118944. The Alzheimer's Disease snRNA-seq and snATAC-seq used in this study are available from GSE174367. The colorectal cancer scRNA-seq data is available from GSE178318. All processed data in this manuscript are available at https://github.com/Starlitnightly/omicverse-reproducibility.

Research involving human participants, their data, or biological material

Policy information about studies with human participants or human data. See also policy information about sex, gender (identity/presentation), and sexual orientation and race, ethnicity and racism.

Reporting on sex and gender	N/A
Reporting on race, ethnicity, or other socially relevant groupings	N/A
Population characteristics	N/A
Recruitment	N/A
Ethics oversight	N/A

Note that full information on the approval of the study protocol must also be provided in the manuscript.

Field-specific reporting

Please select the one below that is the best fit for your research. If you are not sure, read the appropriate sections before making your selection.

- [x] Life sciences
- [ ] Behavioural & social sciences
- [ ] Ecological, evolutionary & environmental sciences

For a reference copy of the document with all sections, see nature.com/documents/nr-reporting-summary-flat.pdf

Life sciences study design

All studies must disclose on these points even when the disclosure is negative.

Sample size	All computational evaluations were repeated multiple times using different random initializations to evaluate algorithmic stability. For most evaluations, we used n=8 repeats which was empirically sufficient to capture the mean and variance of model performance. The only exception was hyperparameter evaluation, where we used n=4 repeats (a minimal for empirical estimation of mean and variance) to reduce computational cost due to the large number of hyperparameter combinations evaluated.
Data exclusions	No data were excluded from analysis.
Replication	All computational experiments were assembled using conda environment configuration files are provided to ensure reproducibility.<br>All attempts at replication were successful.
Randomization	We didn't have control over the experimental design because we utilized pre-existing public datasets. Thus, this is not applicable.
Blinding	We didn't have control over the experimental design because we utilized pre-existing public datasets. Thus, this is not applicable.

Reporting for specific materials, systems and methods

We require information from authors about some types of materials, experimental systems and methods used in many studies. Here, indicate whether each material, system or method listed is relevant to your study. If you are not sure if a list item applies to your research, read the appropriate section before selecting a response.

Materials & experimental systems

n/a	Involved in the study
☒	☐ Antibodies
☒	☐ Eukaryotic cell lines
☒	☐ Palaeontology and archaeology
☒	☐ Animals and other organisms
☒	☐ Clinical data
☒	☐ Dual use research of concern
☒	☐ Plants

Methods

n/a	Involved in the study
☒	☐ ChIP-seq
☒	☐ Flow cytometry
☒	☐ MRI-based neuroimaging
